# Hyperthermia‐induced cytotoxicity and modulation of PD‐L1 and MHC‐I expression in human non‐small cell lung cancer cell lines

**DOI:** 10.1113/EP092791

**Published:** 2025-07-08

**Authors:** Yun‐Chieh Tu, Wei‐Chen Yeh, Yi‐Wei Fang, Ko‐Hsuan Lo, Lei‐Ni Liang, Xu‐Chen Liu, Chia‐Chi Tsai, Chih‐Cheng Cheng, Meng‐Chieh Lin, Hsin‐Hsien Yu, Bor‐Chyuan Su

**Affiliations:** ^1^ School of Medicine, College of Medicine Taipei Medical University Taipei Taiwan; ^2^ School of Pharmacy, College of Pharmacy Taipei Medical University Taipei Taiwan; ^3^ Division of General Surgery, Department of Surgery Wan Fang Hospital, Taipei Medical University Taipei Taiwan; ^4^ School of Medical Laboratory Science and Biotechnology, College of Medical Science and Technology Taipei Medical University Taipei Taiwan; ^5^ Division of General Surgery, Department of Surgery, School of Medicine, College of Medicine Taipei Medical University Taipei Taiwan; ^6^ Department of Anatomy and Cell Biology, School of Medicine, College of Medicine Taipei Medical University Taipei Taiwan

**Keywords:** cytotoxicity, hyperthermia, lung cancer, major histocompatibility complex‐I, programmed death ligand‐1

## Abstract

Hyperthermia has recently been applied to treat human non‐small cell lung cancer (NSCLC). However, the mechanisms underlying cytotoxic sensitivity of NSCLC cells to hyperthermia are not fully understood. In this study, five NSCLC cell lines with different epidermal growth factor receptor (*EGFR*), Kirsten rat sarcoma and tumor protein p53 mutation profiles (A549, H292, H1299, PC9 and H1975) were used to evaluate effects of hyperthermia. All tested cell lines except H1975 were sensitive to hyperthermia‐induced cytotoxicity. Annexin V–propidium iodide double staining, Poly(ADP‐ribose) polymerase (PARP) cleavage and scanning electron microscopy revealed that apoptosis and necrosis were induced by hyperthermia in different lines. Tetramethylrhodamine, ethyl ester analysis further revealed that hyperthermia affected mitochondrial function in the four hyperthermia‐sensitive lines. Transmission electron microscopic analysis revealed degeneration of cristae and ruptured mitochondria upon exposure to hyperthermia. Hyperthermia also caused elevation of reactive oxygen species in sensitive cells. In addition to these effects, hyperthermia impacted cell survival‐related signalling proteins (EGFR, FAK and Akt). In particular, hyperthermia increased phosphorylated EGFR but suppressed total EGFR, phosphorylated Akt and total Akt in sensitive cells. Moreover, hyperthermia could modulate immunomodulatory molecules. Major histocompatibility complex‐I (MHC‐I) and surface programmed death ligand‐1 (PD‐L1) were both elevated by hyperthermia in all tested NSCLC cell lines except PC9. Taken together, our findings provide insights into the potential influence of different somatic mutations in NSCLC cells on hyperthermia‐induced cytotoxicity and regulation of key immunomodulatory molecules.

## Abstract (highlights)

- **What is the central question of this study?**
  The precise mechanisms underlying cytotoxic sensitivity of non‐small cell lung cancer (NSCLC) cells to hyperthermia are not clear.
- **What is the main finding and its importance?**
  Hyperthermia exhibits potent cytotoxic activity in most NSCLC cells, except for cells that harbour the EGFR L858R/T790M mutation.

## 1 INTRODUCTION

Hyperthermia has been used in a wide range of clinical applications, including the treatment of major depressive disorder (Flux et al., 2023), pain (Takahashi et al., 2011) and bacterial infections (Gazel et al., 2024). In addition, hyperthermia has attracted the attention of oncologists owing to its potential anti‐tumour actions (Kok et al., 2020; Lobato et al., 2023). Up to now, several heating technologies have been used in cancer treatments, including electromagnetic heating, hyperthermic perfusion, ultrasound, radiofrequency, microwaves and nanoparticles (Kok et al., 2020). These techniques can be used to heat the tumour to temperatures ranging from 39°C to 100°C, or even higher (Kok et al., 2020), which might directly kill tumour cells (Hou et al., 2014). Of note, the most common and widely accepted target temperature is 43°C (Hou et al., 2014; Su et al., 2024). Intriguingly, hyperthermia not only exhibits broad‐spectrum anticancer activity (Hou et al., 2014; IJff et al., 2022; Shellman et al., 2008) but also shows selective cytotoxicity against cancer cells (Kase & Hahn, 1975). The mechanisms of hyperthermia‐induced cancer cell death have been studied widely in different cellular contexts and might involve DNA damage/repair, protein denaturation, mitochondrial damage, endoplasmic reticulum stress, oxidative stress and activation of p53 (Hou et al., 2014; Kok et al., 2020; Luo et al., 2017; Shellman et al., 2008). In addition to its direct anticancer effects, hyperthermia can sensitize tumour cells to radiotherapy‐ and chemotherapy‐mediated tumour suppression via similarly diverse mechanisms (Dellinger & Han, 2021; Kok et al., 2020; Sharma et al., 2019; Su et al., 2024).

Non‐small cell lung cancer (NSCLC) is the most common type of lung cancer in both males and females (Thai et al., 2021; Zappa & Mousa, 2016). Despite the availability of multiple therapeutic options (surgery, chemotherapy, radiotherapy, targeted therapy and immunotherapy), the therapeutic efficacy of NSCLC treatments remains unsatisfactory. As a result, the estimated 5‐year survival rate of NSCLC patients is <30% (Ganti et al., 2021). Several key gene mutations have been identified in NSCLC, such as epidermal growth factor receptor (*EGFR*), Kirsten rat sarcoma (*KRAS*) and tumor protein p53 (*TP53*) (Thai et al., 2021). These mutations can greatly influence patient prognosis (Marabese et al., 2015; Thai et al., 2021; Wan et al., 2022) and therapeutic efficacy (Liang et al., 2014). For example, NSCLC patients with *EGFR* mutations have better response to chemotherapy, but poorer response to anti‐PD‐1/anti‐programmed death ligand‐1 (PD‐L1) when compared with patients who have *EGFR* wild‐type (WT) NSCLC (Tu et al., 2022). Although platinum‐based chemotherapy exhibits similar therapeutic efficacies in NSCLC patients with *KRAS* mutation and those with *KRAS* WT (Ferrer et al., 2018), mutations in *KRAS G12C* or *TP53* are associated with better objective response and longer progression‐free survival of NSCLC patients receiving anti‐PD‐1/PD‐L1 therapy (Gu et al., 2024; Liu & Gao, 2023). Overall, these studies indicate that patients with different gene mutation profiles can be expected to respond differently to each therapeutic approach.

Recent studies have demonstrated that hyperthermia is a feasible therapeutic approach for NSCLC (Bleehen, 1989; Yang et al., 2019; Zhou et al., 2022), yet the impacts of different gene mutations on hyperthermia‐mediated anti‐NSCLC activity are not fully understood. We hypothesized that hyperthermia might exhibit different cytotoxic activities in NSCLC cells with different mutation profiles. Therefore, we used five NSCLC cell lines with different *EGFR*, *KRAS* and *TP53* mutation profiles and assessed the cytotoxic and mechanistic responses of each cell line to hyperthermia.

## 2 MATERIALS AND METHODS

### 2.1 Reagents

MTS reagent was purchased from Promega (USA). Crystal Violet, phenazine methosulphate (PMS) and propidium iodide (PI) were purchased from Merck (Germany).

### 2.2 Cell lines and cell culture

The A549 and H1975 cell lines were purchased from American Type Culture Collection (USA). H292 cells were purchased from the Bioresource Collection and Research Center (BCRC, Taiwan). The H1299 cell line was a kind gift from Dr Pan‐Chyr Yang (Institute of Biomedical Sciences, Academia Sinica, Taiwan). PC9 cells were a kind gift from Dr Yi‐Ching Wang (Department of Pharmacology, National Cheng Kung University, Taiwan). A549, H1299 and PC9 cells were maintained in Dulbecco's modified Eagle's medium (Thermo Fisher Scientific, USA) supplemented with 10% fetal bovine serum (Peak, USA) and antibiotic–antimycotic (Thermo Fisher Scientific, USA). H1975 and H292 cells were maintained in Roswell Park Memorial Institute (RPMI) (Thermo Fisher Scientific, USA) supplemented with 10% fetal bovine serum and antibiotic–antimycotic (Thermo Fisher Scientific, USA).

### 2.3 Cell viability assay

In order to measure cell viability, the MTS/PMS assay was performed according to the manufacturer's instructions. Cell viability was also assessed by the Crystal Violet assay (Feoktistova et al., 2016). Briefly, cells were seeded in six‐well plates and grown to 90% confluence. The cells were incubated at 37°C or 43°C (hyperthermia) for 1 h, then incubated at 37°C for another 23 h (Su et al., 2024). Thereafter, cells were fixed in glutaraldehyde and stained with Crystal Violet for 30 min. Excessive Crystal Violet was washed off with distilled water. Cell death was also measured by forward scatter (FSC) and side scatter (SSC) features using flow cytometry (Beckman Coulter, USA). Cells that exhibited low FSC and high SSC features were considered dead (Koopman et al., 1994).

### 2.4 Apoptosis, necrosis and cell death analysis

For detection of apoptosis and necrosis, annexin V‐FITC (Biolegend, USA) and PI double staining were performed as previously described (Lin et al., 2024). The intensities of annexin V‐FITC and PI were measured using flow cytometry (Beckman Coulter). Annexin V^+^/PI^−^ (lower right quadrant) and annexin V^+^/PI^+^ (upper right quadrant) cells were defined as apoptotic cells (Cendoroglo et al., 1997). Annexin V^−^/PI^+^ (upper left quadrant) cells were defined as necrotic cells (Cendoroglo et al., 1997). Annexin V^+^/PI^−^, annexin V^+^/PI^+^ and annexin V^−^/PI^+^ cells were defined as dead cells.

The cleavage of Poly(ADP‐ribose) polymerase (PARP) was detected by western blot analysis with an anti‐PARP antibody (Cell Signaling Technology, USA). The detailed procedure is described below (section 2.7). Morphological hallmarks of apoptosis and necrosis were observed by scanning electron microscopy (SEM). The detailed procedure is described below (section 2.5).

### 2.5 Scanning electron microscopy and transmission electron microscopy

For SEM and transmission electron microscopy (TEM), cells were fixed in 2% paraformaldehyde and 2.5% glutaraldehyde. Afterwards, cell pellets were washed with 0.1 M cacodylate and postfixed in OsO_4_. Then, cell pellets were washed with 0.1 M cacodylate three times. The cell pellets were dehydrated through a series of mixed ethanol–water solutions (70%–100% ethanol) and embedded in epoxy resin. Ultrathin sections were observed using a SEM SU3500 (Hitachi, Japan) and a TEM H600 (Hitachi, Japan).

### 2.6 Analysis of mitochondrial membrane potential and reactive oxygen species

To monitor mitochondrial function, cells were loaded with tetramethylrhodamine, ethyl ester (TMRE) (100 nM; ThermoFisher, USA) for 15 min. Then, cells were rinsed with PBS. TMRE intensities were measured using flow cytometry (Beckman Coulter).

In order to measure the levels of intracellular reactive oxygen species (ROS), cells were loaded with 2'7'‐Dichlorodihydrofluorescein diacetate (DCFDA) (10 µM, Merck, Germany). Excessive DCFDA was washed off with PBS three times. DCFDA intensities were measured using flow cytometry (Beckman Coulter).

### 2.7 Western blot analysis

Cells were lysed in RIPA buffer. The cell lysates were then centrifuged at 11269 g at 4°C for 10 min. Supernatants were collected and heated at 100°C for 10 min. Then, protein samples were stored at −20°C before use. Protein samples were separated on a gradient gel and transferred to a polyvinylidene fluoride membrane (Cytiva, USA). The membrane was then incubated with indicated antibodies (Cell Signaling Technology, USA) and corresponding secondary antibodies (Jackson ImmunoResearch, USA). Protein bands were visualized using enhanced chemiluminescence (ECL) horseradish peroxidase (HRP) substrate (Merck Millipore, Germany).

### 2.8 Surface MHC‐I and PD‐L1 analysis

After exposure to hyperthermia, cells were collected and incubated with Alexa Fluor 488‐conjugated anti‐MHC‐I antibody (Santa Cruz Biotechnology, USA) or Alexa Fluor 488‐conjugated anti‐PD‐L1 antibody (Cell Signaling Technology, USA) at 4°C for 1 h. Then, cells were rinsed with PBS. Surface MHC‐I and PD‐L1 expressions were measured using flow cytometry (Beckman Coulter).

### 2.9 Statistical analysis

All experiments were performed in triplicate. Results are expressed as the mean ± SD. Significant differences between indicated groups were identified using Student's unpaired *t*‐test. A value of *p *< 0.05 was considered statistically significant.

## 3 RESULTS

### 3.1 Effects of hyperthermia on the viability of NSCLC cells harbouring different mutation profiles

**FIGURE 1:**
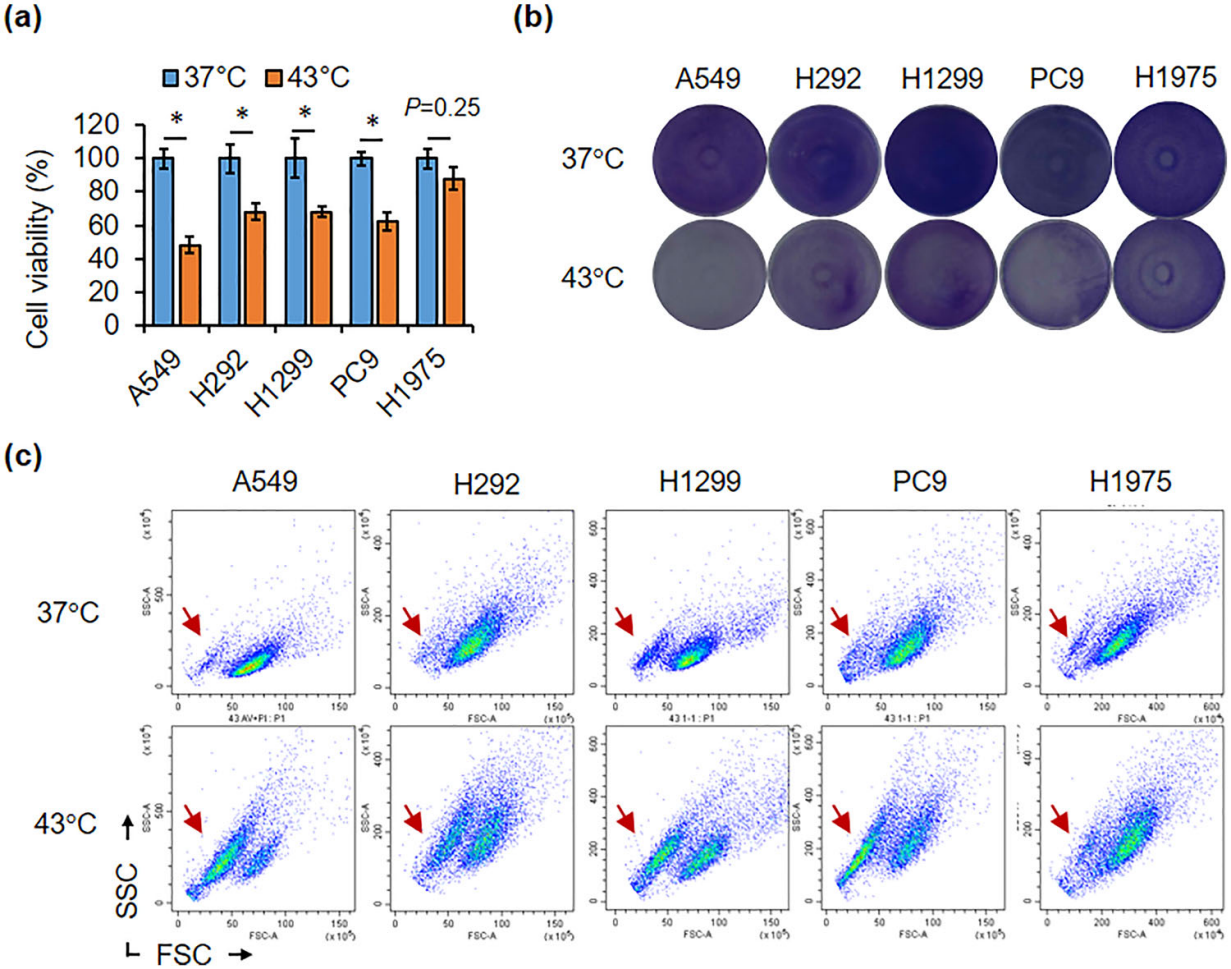
Impact of exposure to hyperthermia on cell viability in NSCLC cells harbouring different mutation profiles. The NSCLC cells were incubated at 37°C or 43°C for 1 h, then incubated at 37°C for another 23 h. Cell viability was assessed by MTS/PMS activity assay (a), Crystal Violet assay (b) and FSC/SSC analysis (c). Red arrows indicate dead cells. ^*^ *p *< 0.05. Abbreviations: FSC, forward scatter; NSCLC, non‐small cell lung cancer; PMS, phenazine methosulphate; SSC, side scatter.

To test how hyperthermia causes cytotoxicity in NSCLC cells with different mutation profiles, five NSCLC cell lines were selected for experiments: A549 cells carry *EGFR* WT, *KRAS* mutation and *TP53* WT; H1299 cells carry *EGFR* WT, *KRAS* WT and *TP53* mutation; H292 cells carry *EGFR* WT, *KRAS* WT and *TP53* WT; and H1975 cells and PC9 cells both carry *EGFR* mutation, *KRAS* WT and *TP53* WT (Table 1). Given that the most commonly used treatment setting for hyperthermia‐based anticancer studies is 43°C for 1 h (Crezee et al., 2021; Su et al., 2024), cells were treated by incubating at 37°C (control) or 43°C (hyperthermia) for 1 h. Then, the cells were incubated at 37°C for another 23 h. Cell viability was assessed with the MTS/PMS activity assay (Fig. 1a), Crystal Violet assay (Fig. 1b) and FSC/SSC analysis (Fig. 1c). The results revealed that hyperthermia treatments effectively reduced cell viability in four of the NSCLC cell lines but not in H1975 cells. The A549 cell line (*EGFR* WT, *KRAS* mutation and *TP53* WT) was the most susceptible to hyperthermia, whereas H1975 cells (*EGFR* mutation, *KRAS* WT and and *TP53* WT) were insensitive to the exposure to hyperthermia.

**TABLE 1:** Characteristics of the non‐small cell lung cancer lines used in this study.

Cell line	Histology	*EGFR*	*KRAS*	*TP53*
A549	Adenocarcinoma	WT	Mutation	WT
H1299	Large cell carcinoma	WT	WT	Mutation
H292	Adenocarcinoma	WT	WT	WT
H1975	Adenocarcinoma	L858R/T790M	WT	WT
PC9	Adenocarcinoma	Exon19 del	WT	WT

### 3.2 Hyperthermia induces apoptosis, necrosis and cell death in NSCLC cells

**FIGURE 2:**
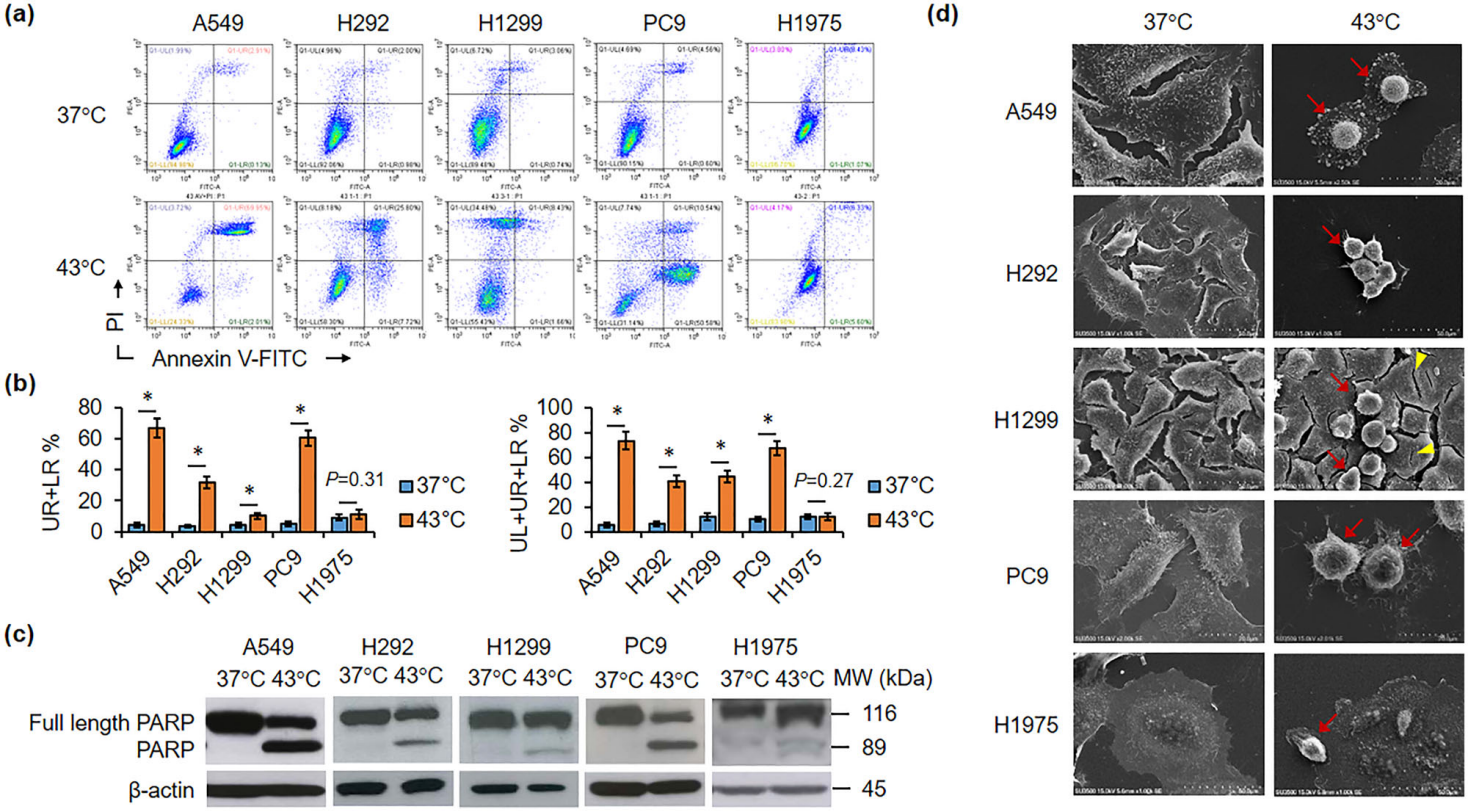
Effects of exposure to hyperthermia on apoptosis/necrosis/cell death in NSCLC cells. NSCLC cells were incubated at 37°C or 43°C for 1 h, then incubated at 37°C for another 23 h. Apoptosis and necrosis were validated by annexin V‐FITC/PI double staining (a,b), cleavage of PARP (c) and SEM (d). Red arrows indicate apoptotic cells. Yellow arrowheads indicate ruptured plasma membrane. ^*^ *p *< 0.05. Abbreviations: NSCLC, non‐small cell lung cancer; PI, propidium iodide; SEM, scanning electron microscopy.

The cytotoxic effects of hyperthermia on NSCLC cells were assessed further using the annexin V/PI double staining assay. Upper right (UR) and lower right (LR) quadrants (annexin V^+^/PI^+^ and annexin V^+^/PI^−^) were regarded as apoptotic cells. The upper left quadrant (UL; annexin V^−^/PI^+^) was regarded as necrotic cells. The UR + LR + UL quadrants were counted together to indicate total cell death. The assay results revealed that hyperthermia induced apoptosis and cell death in A549, H292, H1299 and PC9 cells (Fig. 2a,b). In particular, hyperthermia‐induced necrosis was most prominent in H1299 cells. Notably, H1975 cells were still insensitive to hyperthermia‐induced cytotoxicity, as measured with this assay.

Cleavage of PARP (an apoptosis marker) was also evaluated by western blot analysis. We found that hyperthermia induced PARP cleavage in A549, H292, H1299 and PC9 cells (Fig. 2c). Notably, hyperthermia had no effect on PARP cleavage in H1975 cells.

Next, by performing SEM we assessed whether ultramorphological features of the cells were disrupted. Apoptosis‐associated morphological features, such as cell shrinkage and rounded morphology (red arrows), were observed in hyperthermia‐treated cells (A549, H292, H1299, PC9 and H1975) (Fig. 2d). Although apoptotic cells could be observed in hyperthermia‐treated H1975 cultures, most of cells remained firmly attached to the substrate. Moreover, plasma membrane rupture, a prominent feature of necrosis, was observed in hyperthermia‐treated H1299 cells (yellow arrowheads).

### 3.3 Effects of hyperthermia on mitochondrial function and mitochondrial ultrastructure in NSCLC cells

**FIGURE 3:**
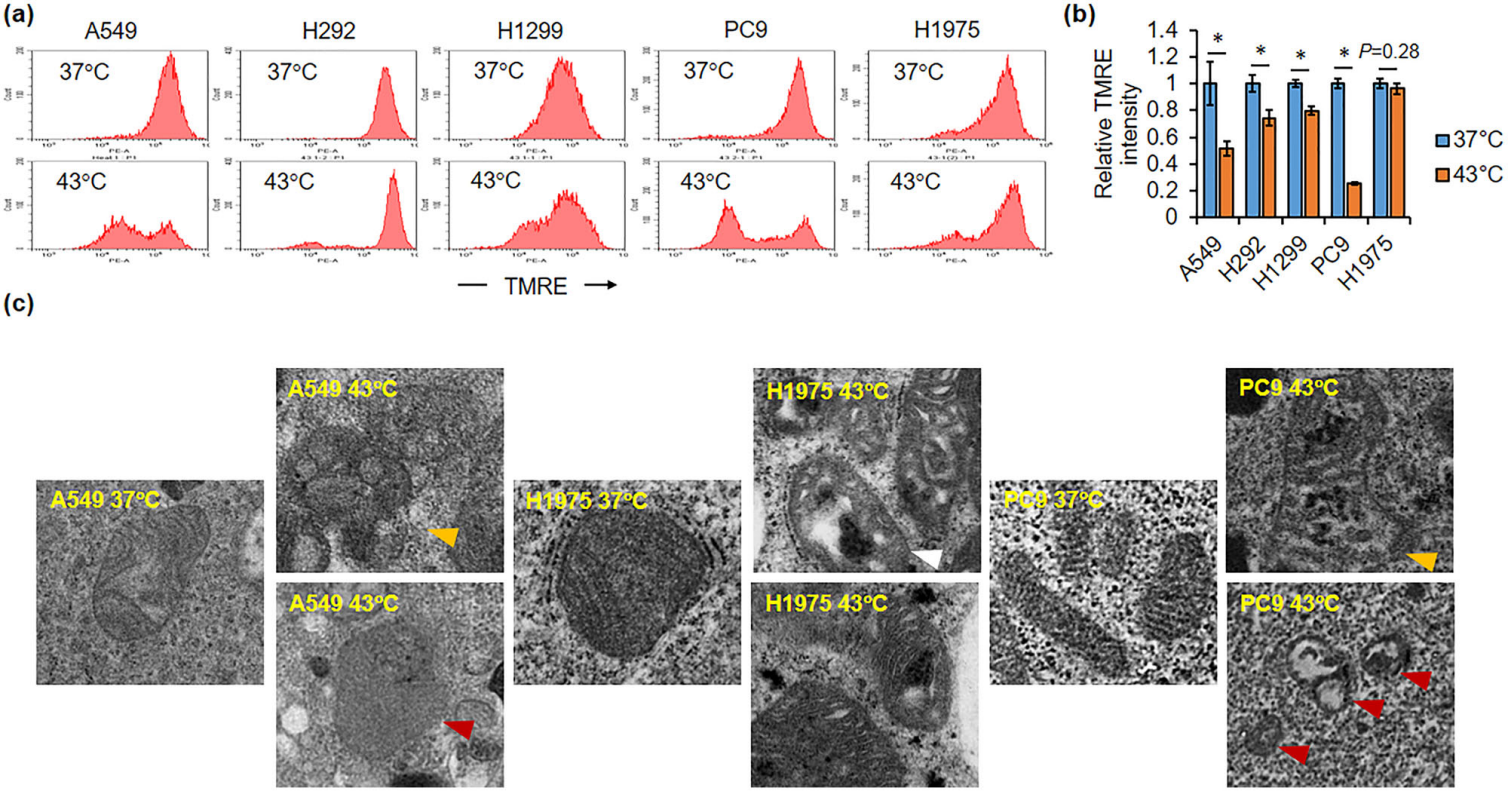
Effects of exposure to hyperthermia on mitochondrial function and ultrastructure. Cells were incubated at 37°C or 43°C for 1 h, then incubated at 37°C for another 23 h. (a,b) The mitochondrial membrane potential was analysed by TMRE staining. (c) Mitochondrial ultrastructure was observed by transmission electron microscopy analysis. ^*^ *p *< 0.05. White arrowhead indicates low‐grade mitochondrial crista degeneration; yellow arrowheads indicate rupture of the mitochondrial outer membrane; and red arrowheads indicate degenerated crista structure.

Given that mitochondria play essential roles in regulating apoptosis and necrosis (Glover et al., 2024), the effects of therapeutic hyperthermia on mitochondrial status were probed. We found that hyperthermia greatly reduced TMRE intensity in four NSCLC cell lines, not including H1975 (Fig. 3a,b). Among the sensitive lines, PC9 was the most vulnerable to hyperthermia‐induced mitochondrial damage. We next investigated the mitochondrial ultrastructure by TEM. Exposure of A549 and PC9 cells to hyperthermia induced degeneration of mitochondrial cristae (red arrowheads) and rupture of the mitochondrial membrane (yellow arrowheads; Fig. 3c). Of note, therapeutic hyperthermia only induced minor alterations to mitochondrial ultrastructure in H1975 cells (Fig. 3c), with a small fraction of mitochondria exhibiting low‐grade cristae degeneration (white arrowhead).

### 3.4 Effects of hyperthermia on ROS and antioxidant protein levels in NSCLC cells

**FIGURE 4:**
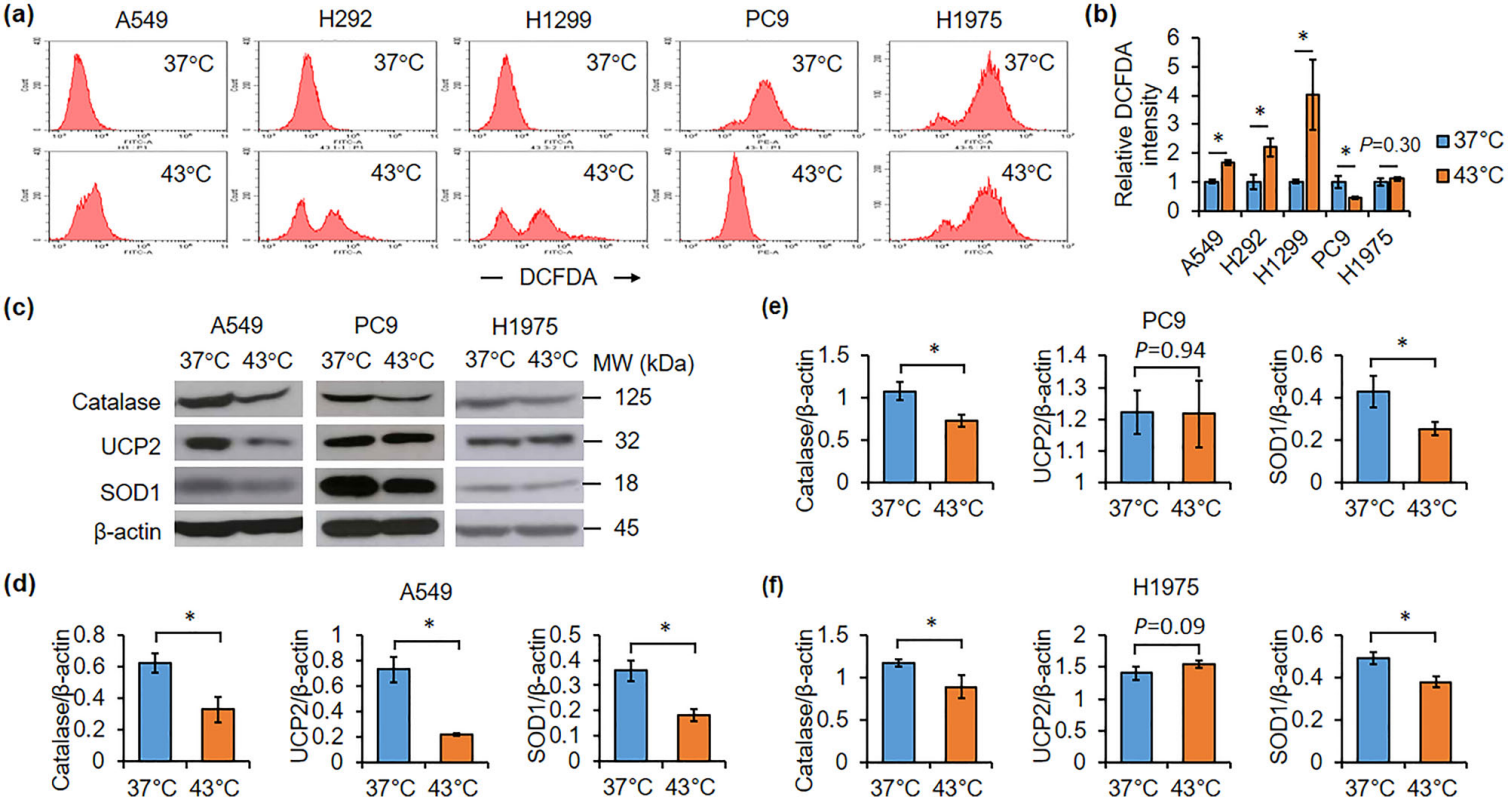
Effects of exposure to hyperthermia on oxidative stress. Cells were incubated at 37°C or 43°C for 1 h, then incubated at 37°C for another 23 h. (a,b) Intracellular ROS were probed by DCFDA. The intensity of DCFDA was measured using flow cytometry. (c–f) Levels of antioxidant proteins (i.e., catalase, UCP2 and SOD1) were validated by western blot analysis. Band intensity was analysed by ImageJ. ^*^ *p *< 0.05. Abbreviations: ROS, reactive oxygen species; SOD1, superoxide dismutase 1; UCP2, uncoupling protein 2.

To measure the effects of hyperthermia on intracellular ROS levels in NSCLC cell lines, the cells were exposed to hyperthermia, followed by quantification of intracellular ROS levels with DCFDA (Fig. 4a,b). Hyperthermia led to elevated intracellular ROS in A549, H292 and H1299 cells, whereas ROS were reduced by hyperthermia in PC9 cells. Notably, hyperthermia had no effect on intracellular ROS levels in H1975 cells.

We also assessed the levels of antioxidant proteins, including catalase, uncoupling protein 2 (UCP2) and superoxide dismutase 1 (SOD1). Western blot analyses revealed that hyperthermia suppressed all examined antioxidant proteins in A549 cells (Fig. 4c,d). Only catalase and SOD1 were suppressed by hyperthermia in PC9 cells (Fig. 4c,e). Interestingly, although hyperthermia had no effect on intracellular ROS levels in H1975 cells, both catalase and SOD1 were slightly suppressed by the hyperthermia treatment (Fig. 4c,f).

### 3.5 Effects of hyperthermia on the EGFR/FAK/Akt signalling in NSCLC cells

**FIGURE 5:**
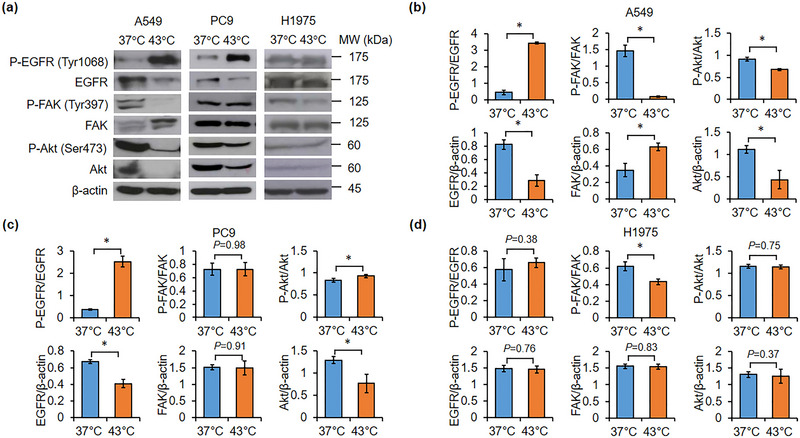
Impacts of therapeutic hyperthermia exposure on EGFR/FAK/Akt signalling. (a–d) Cells were incubated at 37°C or 43°C for 1 h, then incubated at 37°C for another 23 h. Levels of P‐EGFR (Tyr1068), EGFR, P‐FAK (Tyr397), FAK, P‐Akt (Ser473), Akt and β‐actin were validated by western blot analysis. Band intensity was analysed by ImageJ. ^*^ *p *< 0.05. Abbreviations: EGFR, epidermal growth factor receptor.

Given that EGFR/FAK/Akt signalling is essential for NSCLC cell growth, survival, and sensitivity to cytotoxic agents and stress (Howe et al., 2016; Rice et al., 2019), we next tested the effects of hyperthermia on EGFR/FAK/Akt signalling using western blot analysis. The results revealed that phosphorylated EGFR (Tyr1068) and FAK were elevated by hyperthermia in A549 cells, whereas total EGFR, phosphorylated FAK (Tyr397), phosphorylated Akt (Ser473) and total AKT were suppressed (Fig. 5a,b). Hyperthermia induced similar effects on phosphorylated EGFR, total EGFR, phosphorylated Akt (Ser473) and total Akt in PC9 cells (Fig. 5a,c). However, hyperthermia had no effect on phosphorylated FAK (Tyr397) and total FAK. Notably, most of the examined signalling proteins were not altered by hyperthermia in H1975 cells; only phosphorylated FAK (Tyr397) was suppressed in this line (Fig. 5a,d).

### 3.6 Hyperthermia regulates MHC‐I and PD‐L1 levels

**FIGURE 6:**
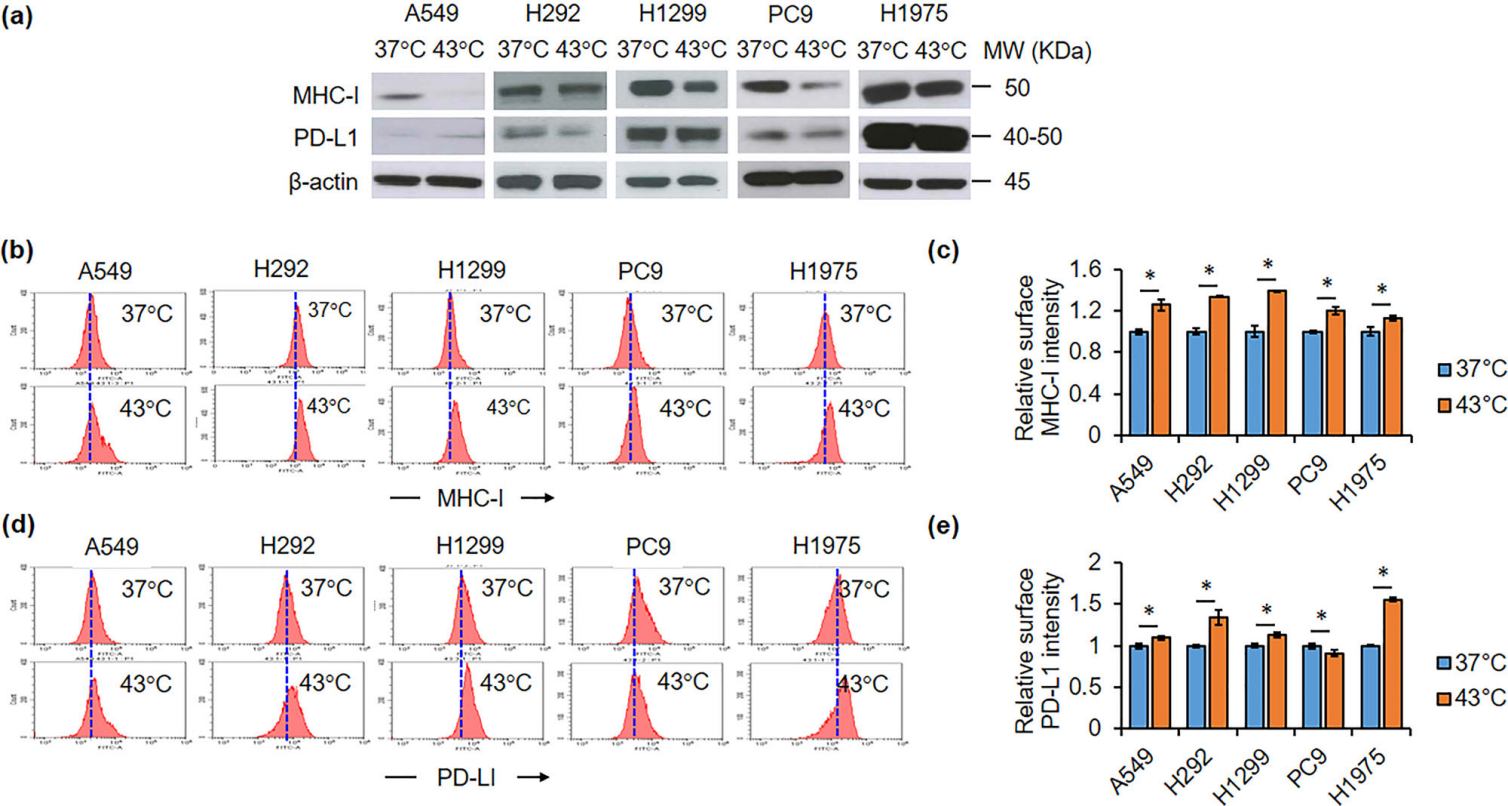
Impacts of therapeutic hyperthermia exposure on MHC‐I and PD‐L1. Cells were incubated at 37°C or 43°C for 1 h, then incubated at 37°C for another 23 h. (a) Total protein levels of MHC‐I and PD‐L1 were detected by western blot analysis. (b–d) Surface MHC‐I (b,c) and surface PD‐L1 (d,e) were probed by Alexa Fluor 488‐conjugated MHC‐I and Alexa Fluor 488‐conjugated PD‐L1 antibodies, respectively. Surface levels of MHC‐I and PD‐L1 were measured using flow cytometry. ^*^ *p *< 0.05. Abbreviations: MHC‐I, major histocompatibility complex‐I; PD‐L1, programmed death ligand‐1.

Expression of MHC‐1 and PD‐L1 can be affected dynamically by environmental stress and chemotherapeutic agents (Lu et al., 2020; Mortezaee et al., 2023). Moreover, the expression of these two proteins is a determinant of the efficacy of anti‐PD‐1/PD‐L1 therapy (Li et al., 2022; Shklovskaya et al., 2020). Given that the potential effects of hyperthermia on MHC‐I and PD‐L1 expression in NSCLC cells remained unclear, we began by testing how hyperthermia affects MHC‐I and PD‐L1 expression at total protein levels. The results demonstrated that hyperthermia suppressed MHC‐I expression in all tested cell lines, but the suppressive effects were relatively minor in H292 and H1975 cells (Fig. 6a). Hyperthermia elevated PD‐L1 expression in A549 cells, whereas the treatment suppressed PD‐L1 expression in H292 and PC9 cells. In contrast, hyperthermia did not alter the levels of PD‐L1 in H1299 and H1975 cells. We next assessed the surface levels of MHC‐I by flow cytometry. We found that hyperthermia elevated surface MHC‐I levels in all NSCLC cell lines (Fig. 6b,c). Measuring the surface PD‐L1 levels revealed that hyperthermia increased surface PD‐L1 in four of the NSCLC cell lines, excluding PC9 (Fig. 6d,e). In PC9 cells, the surface PD‐L1 level was slightly suppressed by hyperthermia.

## 4 DISCUSSION

Several gene mutations are correlated with prognosis and therapeutic efficacy in NSCLC, including *EGFR*, *KRAS* and *TP53* (Thai et al., 2021). Moreover, hyperthermia therapies are being used increasingly to treat lung cancer in applications such as intrapleural hyperthermic chemotherapy (Shin et al., 2024), lung tumour ablation (Hung et al., 2024), computed tomography‐guided microwave ablation (Hu et al., 2024), photothermal therapy (Yilmazer et al., 2023), radiofrequency hyperthermia (Tian et al., 2023) and microwave ablation (Wang et al., 2023). However, the benefits of hyperthermia‐based therapies for lung cancer remain controversial (Liu et al., 2022; Zhou et al., 2022). Furthermore, it remains unclear whether common gene mutations might influence the outcomes of hyperthermia treatment.

In this study, we describe how hyperthermia causes cytotoxicity in a panel of NSCLC cell lines with different gene mutations. Interestingly, hyperthermia caused cytotoxicity in four of the five tested NSCLC cell lines, but it did not appear to have strong effects on H1975 cells. Furthermore, hyperthermia mainly induced necrosis in H1299 cells (*EGFR* WT, *KRAS* WT and *TP53* mutation), but it induced apoptosis in A549 cells (*EGFR* WT, *KRAS* mutation and *TP53* WT), H292 cells (*EGFR* WT, *KRAS* WT and *TP53* WT) and PC9 cells (*EGFR* mutation, *KRAS* WT and *TP53* WT). Although both H1975 and PC9 cell lines both carry *EGFR* mutations, the mutant subtypes are different. H1975 carries an *EGFR* L858R/T790M double mutation, whereas PC9 cells harbour an *EGFR* exon 19 mutation. Surprisingly, PC9 cells were as sensitive to hyperthermia as *EGFR* WT cell lines, but H1975 was resistant to hyperthermia‐mediated cytotoxicity. Based on these findings, we can speculate that hyperthermia might not be an efficient treatment in NSCLC patients with *EGFR* L858R/T790M double mutation. This speculation is in line with the results of one previous study that showed that the clinical benefits of hyperthermia are more prominent in NSCLC patients without *EGFR* mutations (Zhou et al., 2022). Nevertheless, the authors of that study did not discuss the potential differences in hyperthermia outcomes for different *EGFR* mutation subtypes. L858R/T790M is not considered to be a common mutation for *EGFR* (Tokumo et al., 2006). Rather, the most common type of *EGFR* mutation is exon 19, which accounts for 46%–76% of *EGFR* mutation (Kaler et al., 2023). Given that hyperthermia caused cytotoxicity in PC9 cells carrying the exon 19 mutation, we conclude that hyperthermia might still be suitable for the majority of NSCLC patients with *EGFR* mutation.

Our analysis revealed that mitochondrial dysfunction was induced by hyperthermia in all tested NSCLC cell lines except H1975 (Fig. 3). Among the responding cell lines, PC9 was the most sensitive to hyperthermia‐induced mitochondrial dysfunction. Analysis of the ultrastructure revealed that hyperthermia causes either cristae degeneration or rupture of the mitochondrial membrane in hyperthermia‐sensitive cells (A549 and PC9), whereas the treatment induces only slight cristae degeneration in hyperthermia‐insensitive cells (H1975) (Fig. 3). Furthermore, hyperthermia elevates intracellular ROS and reduces antioxidant proteins in hyperthermia‐sensitive cells (Fig. 4a,b). Notably, intracellular ROS were reduced by hyperthermia in PC9 cells. Given that ROS are products of mitochondrial respiration (Quintana‐Cabrera et al., 2021), we speculate that hyperthermia might cause more severe suppression of mitochondrial respiratory activity in PC9 cells than in other cell lines.

In hyperthermia‐sensitive cells (A549 and PC9), phosphorylation of EGFR was induced by hyperthermia (Fig. 5). Similar findings were reported by Thompson et al. (2018), who demonstrated that EGFR and mesenchymal‐epithelial transition (MET) signalling were activated by heat stress in hepatocellular carcinoma cell lines (Thompson et al., 2018). They also found that inhibition of EGFR signalling via erlotinib (US Food and Drug Administration‐approved EGFR inhibitor) significantly enhanced heat stress‐induced cytotoxicity, suggesting that increased phosphorylation of EGFR might serve as a protective mechanism to counteract detrimental hyperthermia‐induced effects. Furthermore, we found that the phosphorylation of EGFR was not enhanced in H1975 (hyperthermia‐insensitive) cells (Fig. 5). Therefore, the combination of erlotinib and hyperthermia might be a feasible strategy to enhance the anticancer activity in NSCLC tumours carrying the EGFR L858R/T790M mutation. A previous study showed that Akt is required for hyperthermia‐induced activation of hypoxia‐inducible factor 1α in NSCLC cells. In addition, hypoxia‐inducible factor 1α activation counteracts hyperthermia‐induced suppression of cell proliferation (Wan & Wu, 2016). We found that both phosphorylated Akt and total Akt levels were lowered by hyperthermia treatment in hyperthermia‐sensitive cells (A549 and PC9), whereas this inhibitory effect was not observed in hyperthermia‐insensitive cells (H1975) (Fig. 5). Therefore, it is plausible that the Akt–hypoxia‐inducible factor 1α signalling axis might play a role in hyperthermia resistance and that suppression of this signalling axis might enhance hyperthermia‐mediated cytotoxicity in H1975. Intriguingly, among hyperthermia‐sensitive cells (A549 and PC9) and hyperthermia‐insensitive cells (H1975), H1975 cells were previously shown to have the lowest basal levels of phosphorylated Akt (Pratilas et al., 2008). These results suggest that the basal level of phosphorylated Akt might not be a major determinant of hyperthermia sensitivity in NSCLC cells. Nevertheless, the exact roles of phosphorylated EGFR and phosphorylated FAK in hyperthermia‐induced cytotoxicity require further investigation.

In addition to enhancing chemotherapy‐ and/or radiotherapy‐induced cytotoxicity (Shin et al., 2024; Son et al., 2019), hyperthermia also has immunomodulatory effects in the tumour microenvironment (Li et al., 2020; Skitzki et al., 2009). For instance, hyperthermia promotes CD8 T‐cell differentiation and enhances their tumour‐killing ability (Mace et al., 2012). Moreover, hyperthermia elevates PD‐L1 expression in tumours (Li et al., 2020), and patients with higher tumour PD‐L1 expression typically have better responses to immune checkpoint inhibitors (ICIs) (Fundytus et al., 2021). In our study, we found that hyperthermia increases cell surface PD‐L1 in most of the tested cell lines (except PC9; Fig. 6). Intriguingly, hyperthermia did not cause cytotoxicity in the H1975 cell line, but the treatment induced a robust elevation of surface PD‐L1 expression in these cells. Hyperthermia also increased surface MHC‐I in all tested cell lines. MHC‐I is an essential molecule that presents tumour neoantigens to T cells, and it is silenced in most cancers (Taylor & Balko, 2022). Given that MHC‐I‐mediated tumour recognition is the first step for CD8 T‐cell‐mediated tumour suppression (Wu et al., 2023), its expression is also correlated with the efficacy of ICIs (Taylor & Balko, 2022). Previous work has demonstrated that NSCLC patients with *EGFR* mutations generally exhibit poor response to ICIs (Qiao et al., 2021; To et al., 2021) owing to weak immunogenicity of the tumour cells (low expression of MHC‐1 and PD‐L1) (Gettinger et al., 2018; To et al., 2021). Thus, hyperthermia might be able to improve the response to ICIs by increasing the immunogenicity of NSCLC tumours.

Our results suggest that hyperthermia exhibits potent cytotoxic activity in most NSCLC cells, except for cells that harbour the *EGFR* L858R/T790M mutation. Nevertheless, hyperthermia might create favourable conditions for response to ICIs by increasing surface PD‐L1 and surface MHC‐I expression in all tested cell lines. Thus, our findings demonstrate that hyperthermia might exert anticancer activity via multiple mechanisms.

## AUTHOR CONTRIBUTIONS

This study was performed in Bor‐Chyuan Su's Laboratory at the Taipei Medical University. Yun‐Chieh Tu, Wei‐Chen Yeh and Yi‐Wei Fang conceived and designed the experiments. Ko‐Hsuan Lo, Lei‐Ni Liang and Xu‐Chen Liu acquired the data. Chia‐Chi Tsai, Chih‐Cheng Cheng and Meng‐Chieh Lin analysed the data. Hsin‐Hsien Yu and Bor‐Chyuan Su interpreted the data and drafted the manuscript. All authors approved the final version of the manuscript and agree to be accountable for all aspects of the work in ensuring that questions related to the accuracy or integrity of any part of the work are appropriately investigated and resolved. All persons designated as authors qualify for authorship, and all those who qualify for authorship are listed.

## CONFLICT OF INTEREST

None declared.

## Data Availability

The raw data supporting the conclusions of this article will be made available by the authors without undue reservation.
